# Experiences of infertile women pursuing treatment in Kenya: a qualitative study

**DOI:** 10.1186/s12905-022-01950-4

**Published:** 2022-09-02

**Authors:** Anne Njogu, Julius Njogu, Albanus Mutisya, Yang Luo

**Affiliations:** 1 Xiangya School of Nursing, Central South University, Changsha, 410013 Hunan China; 2 Jomo Kenyatta University of Agriculture and Technology (JKUAT)/Kenya Medical Research Institute (KEMRI), Nairobi, Kenya; 3 Jomo Kenyatta University of Agriculture and Technology (JKUAT), Juja, Kenya

**Keywords:** Infertility, Women, Qualitative, Kenya, Africa

## Abstract

**Background:**

The infertility treatment process is associated with various psychological, physical, social, moral, and financial challenges, especially for women. The women are likely to report low marital satisfaction and emotional distress due to fertility treatment demands. This study explored how infertile women described their treatment experience and how they coped with treatment demands as they underwent treatment at three gynecology outpatient clinics in Kenya.

**Methods:**

A qualitative phenomenological research design was used to analyze and describe women’s fertility treatment experiences. The data were collected through semi-structured in-depth interviews with 33 infertile women selected purposively. Trustworthiness of the findings was ensured using Guba and Lincoln’s criteria. The recorded interviews were transcribed verbatim and then analyzed using reflective thematic analysis, developed by Braun and Clarkes.

**Results:**

Three themes and 13 sub-themes related to women’s fertility treatment experiences and coping strategies were identified. The theme challenges encountered during fertility treatment have three sub-themes: emotional distressing, physical pain, and financial constraining. Theme impacts of fertility treatment on relationships have three sub-themes: relationship with their husband, relationship with their family, and relationship with their friends. Finally, coping with fertility treatment has six sub-themes: religious practices and personal faith, giving in to feelings, shifting focus, taking a break, staying with their relative’s children, and receiving support from others.

**Conclusion:**

The experiences of women undergoing treatment are multi-dimensional. Therefore, incorporating psychosocial interventions or counseling into the fertility treatment routine with National Health Insurance Fund cards may reduce the treatment burden, improving women’s psychological well-being and relationships with their husbands, family, and friends.

## Introduction

Childbearing and raising children are considered significant events in the life of most couples worldwide, particularly in Africa, of which Kenya is no exception. It is widely accepted that human life reaches completeness through the birth of a child since the birth of a child is believed to fulfill the individual need for reproduction [1]. Thus, when a couple fails to bring forth a clinical pregnancy after 12 months or more of regular unprotected sexual intercourse is diagnosed with infertility. Voluntary childlessness is rare in Kenya because parenthood is the ultimate purpose of marriage, and marriage without children is deemed incomplete [2]. Primary infertility, among types of infertility, refers to a couple that has never conceived, and secondary infertility refers to a couple who fails to achieve a clinical pregnancy despite being able to previously [3]. Infertility affects all individuals across gender, nationality, ethnicity, race, religion, and social-economic status. It is estimated that worldwide, 8–12% of couples experience fertility problems during their reproductive years, most of whom are residents of developing countries [3]. Although reliable data do not exist in most African countries, more than 30% of couples are estimated to suffer from infertility [4]. The prevalence of infertility in Kenya is still uncertain. However, a cross-section by Murage et al. [5] found that 26.1% of reproductive health consultations had delayed fertility, with 50% attributed to tubal factors and 15% to male characteristics.

In the quest for parenthood, 50% of infertile couple seek medical assistance, which the initial treatment include medication, surgery, treating underlying medical or congenital cause, and lifestyle modification. If these first-line treatments fail or are deemed inappropriate, about 3% of these couples are recommended to use assisted reproductive technologies (ARTs) [6]. ARTs treatment involves in-vitro handling of human oocytes and sperm or embryos to achieve pregnancy with the expectation of artificial insemination [7]. In-vitro fertilization (IVF), intracytoplasmic sperm injection (ICSI), Gamete Intra Fallopian Transfer (GIFT), Tubal Embryo Transfer (TET), and Zygote Intra Fallopian Transfer (ZIFT) are some examples of ARTs interventions [7]. The ARTs have contributed to about 1–4% of the world population in developed nations. These figures are expected to continue to increase [8]. In recent years, there has been enormous demand for ARTs services in developed and developing countries. The ARTs services are skewed towards high-income countries, while in low-middle-income countries, they are few and restricted to private settings [9]. After the first successful case of IVF in 2006 in Kenya, several other clinics have commenced ARTs services to couples with some degree of success [5].

A shred of growing evidence recognizes that infertility and its treatment are enormous stressful to couples, particularly women. The infertility diagnosis can result in shame, fear, anger, anxiety, sadness, guilt, and loss of control [10, 11]. More often, infertile couples experience marital and sexual satisfaction deterioration because they only concentrate on the biological aspect of the fertility of sexual intercourse, neglecting the pleasure and emotional bonding aspects [12]. ARTs can lead to physical, psychological, financial, and moral challenges. For example, during the treatment, women must adhere to the strict protocol, including ovulation induction, oocyte retrieval, oocyte insemination, and embryo transfer to the uterus. Attend numerous treatment appointments, investigations (blood sample collection and ultrasound scanning), and multiple injections, which may significantly interrupt her daily routines, such as work. After embryo implantation, the women must wait two to three weeks to know whether they are pregnant, which is considered the most stressful phase of treatment [10, 13]. Moreover, ARTs are expensive and require couples to make difficult decisions such as when to start or stop the treatment, how many embryos to transfer, or choosing eggs or donors [12, 14]. Furthermore, some women may reject ARTs treatments for religious reasons as they may consider them morally unacceptable because fertilization occurs outside the body [15].

Given the high value for children in many Kenyan communities and the problems associated with infertility and its treatment as mentioned above, which may overwhelm the couples’ or individuals’ coping skills and social support. It was essential to raise pertinent questions such as:

1. How do Kenyan women pursuing fertility describe their experience?
2. How do Kenyan women faced with infertility cope with treatment problems?

These are the main questions that this study sought to address by exploring how infertile women described their treatment experience and how they coped with treatment demands as they underwent treatment at three gynecology outpatient clinics in Kenya.

## Methods

### Design

A descriptive phenomenological with thematic analysis approach was employed to understand better the experiences of infertile women seeking treatment at three government gynecology outpatient clinics: Kenyatta National Hospital, Thika Level 5 Hospital, and Kiambu Level 5 hospital in Kenya. These hospitals treat individuals experiencing primary or secondary infertility; although they do not offer ARTs, those women who need these services are referred to private hospitals. A qualitative descriptive approach was used because it was most appropriate to explore the experiences of infertile women pursuing treatment regarding how they describe, react, understand and cope with their experiences.

### Study population and sampling strategy

The study population was mainly infertile women seeking treatment at Kenyatta National Hospital, Thika Level 5 Hospital, and Kiambu Level 5 hospital in Kenya. Women were selected through purposive sampling with maximum variation, meaning that the researchers identified and chose participants who could provide extensive information about the phenomenon. In this study, the phenomenon was the experience of women pursuing fertility treatment. Maximum variation was ensured by sampling different hospitals and selecting women of different ages, education, marital status, infertility duration, number of children, previous abortions, time spent undergoing treatment, cause of infertility, and current treatment.

Data collection was carried out from October 2021 to January 2022. During this period, trained nurses working in the three gynecology clinics identified and approached potential participants. Those women who met the inclusion criteria and willing to participate were given informed consent forms to read and familiarize themselves with the study. The women were informed that participation was voluntary and was free to withdraw at any time during the study without their care being affected. They were also assured of their confidentiality and were told this was a minimal potential study with no long-term effects [16]. Participants joined the study after providing written informed consent. The time and date of the interview were arranged according to participant preference.

### Inclusion and exclusion criteria

Women were included in this study if they were aged 20–44 years, (2) diagnosed with infertility, (3) undergoing treatment, (4) and able to follow the instructions, and (5) had mobile phone access. Women were excluded if they (1) had unstable mental conditions during the study period and (2) could not read or speak English and Kiswahili.

### Data collection procedure

A semi-structured interview guide was developed based on the study objectives and a review of relevant literature. The interview guide consisted of two parts. Session A consisted of 10 questions related to the social-demographic and clinical characteristics of the participants. Session B consisted of six questions to elicit information on the experiences of infertile women pursuing treatment regarding how they describe, react, understand and cope with their experiences. For content validation, the interview guide was reviewed by a gynecologist, and a qualitative research expert and pilot tested before interviews (see Table 1).

**Table 1:** The interview topic guide

Title: “Experiences of infertile women pursuing treatment in Kenya”	
Introduction	Study aims Queries and clarifications
Session A	*Personal data* Please tell me about yourself: age, occupation, education, marital status, duration of infertility (years), number of living children, previous abortion, time spent undergoing treatment (years), cause of infertility, and current treatment
Session B	*Experiences of infertility treatment* How did you know you had a problem with getting pregnant? Could you share how you felt when you were told you have infertility? Could you briefly describe the fertility treatment you are currently undergoing? Could you tell me about your fertility treatment experience? Please describe how infertility treatment has affected your relationship with your husbands, family members, others, or daily activities?
	*Coping strategies utilized while pursuing treatment* Please, tell me how you cope with the infertility treatment experiences?

The first author (AN) conducted the individual in-depth semi-structured interviews under the supervision of a woman health specialist (YL). Based on the women’s preferences, 30 interviews were conducted through Zoom, while three were conducted by telephone because the participants had no access to smartphones or computers. Since study interviews were conducted virtually or by phone, women were requested to choose a quiet private room. The interviews were conducted in English and Kiswahili depending on women’s preferences, and most women preferred speaking in English. The first author (AN) is a female Ph.D. student in Maternal Child Health Nursing with an extensive qualitative research background. She completed her qualitative research coursework during the first year of her Ph.D. program. A total of 35 women were contacted, and all agreed to participate, but two declined to participate due to privacy concerns. Therefore, 33 in-depth interviews were conducted. However, data saturation was reached after 30 interviews; the first author (AN) conducted three more interviews to ensure data saturation, meaning that no new themes or information emerged from the last few interviews.

Before the interview, the women were informed how the interview would be conducted and that the interview would be audio-recorded. Oral consent was obtained from the study participants. The women were asked for their personal information, including age, occupation, education, marital status, duration of infertility (years), number of living children, previous abortion, time spent undergoing treatment (years), cause of infertility, and current treatment. Subsequently, general and open-ended questions were asked using an interview guide. For those participants who declined audio-recording, their interviews were handwritten. The probing questions such as how, what do you mean, and please explain more were used to elicit further information. Detailed notes were taken during the interviews. The interviews lasted between 45 to 60 min. All interview transcripts were sent to women via E-mail or WhatsApp for feedback. All participants reported that the transcripts reflected their interview responses regarding their experiences and coping strategies and suggested no corrections or changes.

### The position and affiliations of the authors

In a qualitative study, researchers recognize the importance of reflexivity and transparency about their preoccupations and assumptions [17]. The first author (AN) was born and raised in Kenya and moved to China to pursue her Doctorate studies. She has experience in obstetrics and gynecology as a nurse. Notably, AN did not have any prior relationship with study participants. The second author (JN) has an experience in nursing, public health, and reproductive health. He is currently pursuing his Doctorate in Public health in Kenya. The third author (MK) is a senior nursing lecturer at a Kenyan university and has an extensive experience in qualitative research and mental health. The fourth author (YL) is a senior nursing lecturer in China and a women’s health expert. Her positionality as an outsider- a foreign researcher- made her add insightful observations during the interview and data analysis process.

### Data analysis

**Fig. 1:**
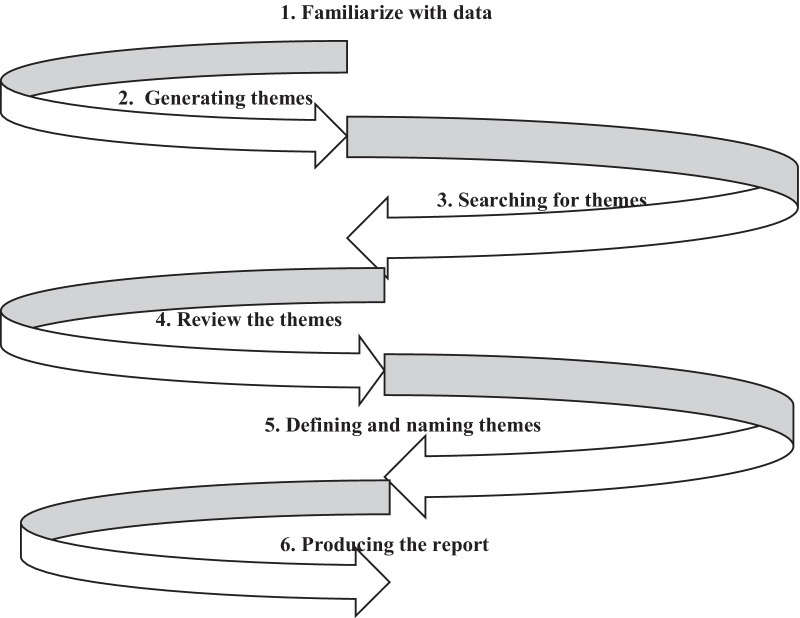
Six phases of reflective thematic analysis

The audio-recorded interviews were transcribed verbatim in the original languages by two authors (AN and JN). The third author (MK) checked the accuracy of the transcripts. The four Kiswahili transcripts were translated into English and back-translated to Kiswahili to check for translation accuracy. The translation was done by two authors (AN and JN), fluent in the two languages, and used consensus to solve disagreements. Software coding NVivo 12 was used to facilitate data management and coding. The data were analyzed according to six phases of reflective thematic analysis described by Braun and Clarkes [18] (see Fig. 1). First, two authors (AN and JN) read each transcript several times to fully understand its content. Second, the AN and JN independently coded the whole data set. Phrases or sentences that described experiences of infertile women pursuing fertility treatment and how they coped with their experiences were labeled with a short description of the content. Third, AN, JN, and MK agreed to combine codes to generate data-derived themes. After generating the initial themes, AN and JN returned to the dataset to determine whether the themes were data-driven, made sense, overlapped, told a convincing story, answered the research questions, or were other themes within the data. Next, all authors defined and named the generated themes, as shown in Table 2. They also assessed identified themes to determine whether data sufficiently supported them. As for producing the report, we adhered to the Standards for Reporting Qualitative Research guidelines (SRQR) [19]. After themes were generated, we conducted member checking, a qualitative research method in which research participants verified the authenticity of qualitative results. Participants confirmed that the generated themes reflected their opinion regarding how they described fertility treatment experiences and coped with their experiences. Notably, codes, subthemes, and themes were arrived at through consensus by all authors. An example of the reflective thematic analysis is shown in Table 2.

**Table 2:** Reflective thematic analysis: from codes to themes

Codes	Sub-themes	Themes
Constantly worried and anxious about treatment outcome	Emotional distressing	Challenges encountered during fertility treatment
Depressed about visiting different hospitals and gynecologists		
Going through the treatment is a lonely experience		
Progesterone shots are painful as they are injected deep into the muscle	Physical pain	
Egg retrieval was the most brutal and most painful procedure		
Experience severe bloating and headache after taking fertility medication		
Applying for a loan to pay for my treatment	Financial constraining	
Asking our family and friends for money to pay for surgery		
Only can access treatment because of an NHIF card		
Loss of sexual interest	Relationship with their husband	Impacts of fertility treatment on relationships
He started drinking excessively and became verbally abusive		
He became work alcoholic		
Become more emotionally connected (Positive effect)		
Feeling hopeful again about becoming parents (Positive effect)		
The mother-in-law is not happy	Relationship with their family	
Intrusive questions about the treatment process		
Offering unnecessary advice		
Lack of closure of treatment journey due to perceiving pressure and fear of their children being stigmatized		
Very disappointed with their best friend	Relationship with their friends	
Loss of friends during the treatment process		
Some friends ask intrusive questions about treatment		
Reading the Bible and praying	Religious practices and personal faith	Coping with fertility treatment
Believing in Allah		
Crying, especially during menses	Giving in to feelings	
Neglect doing their daily activities such as taking a shower		
Indulging themselves by buying new outfits and bags or dinner		
Deciding to pursue higher education	Shifting focus	
Involved more in voluntary work		
Taking a break for two years from treatment	Taking a break	
Taking a break for some months to rest		
Staying with nieces who helped with simple chores	Staying with their relative’s children	
Staying with nephews to create a complete family feeling		
The husband accompanied his wife during a hospital appointment	Receiving support from others	
Mother is very supportive in that she gives advice and encourages during the treatment process		
Friends have been supportive during the treatment process		

### Trustworthiness

To ensure the trustworthiness of the study findings, we used Guba and Lincoln’s criteria of credibility, transferability, dependability, and confirmability [20]. Credibility was achieved through member check, involvement of all authors in the data analysis process, the credibility of the authors and their professional background, reflexivity of authors, searching for disconfirming evidence, and prolonged engagement with data. Transferability was enhanced through purposive sampling with maximum variation, providing a detailed description of participants and the research process and continuously returning to the data. Dependability was achieved through accurate translations and transcription of interviewers and having three external qualitative research experts review the interview guide, raw data, and observational notes. Confirmability was achieved by maintaining a reflective journal during the research process, where the authors documented their reflexivity, bracketing, or relevant events during the study.

### Ethical considerations

The study was approved by the Xiangya Nursing School of Central South University Ethical Review Board, the National Commission for Science, Technology, and Innovation (NACOSTI), and the Kenyatta National Hospital-University of Nairobi Ethics Research Committee. Additional approval was obtained from the respective county and sub-county commissioners, the ministry of health in the respective countries, and research site hospitals. All women consented to participate and allowed the researchers to make follow-up contact to confirm the components and interpretation of the data analysis. All methods were carried out following relevant guidelines and regulations.

### Study results

The 33 women interviewed were aged between 21 and 44 years; 14 women were employed, 12 were entrepreneurs, and seven were housewives. Three women had primary education, 11 had secondary education, and 18 had diploma certificates and above. Twenty-three women were married, four were living with a male partner, four were separated, and two were divorced. The duration of infertility ranges from 2–14 years. One woman had two living children, 14 had one child, and the remainder had no child. Nine women had a history of abortion ranging between 1–3 times. The time spent undergoing treatment was between 1–10 years. Seventeen women indicated female factor infertility; six reported male-factor, five said both female and male-factor, and five reported their infertility was unexplained. Regarding the type of treatment, nine women reported receiving medical treatment, 14 were undergoing surgical treatment with their partners, and ten were pursuing ART. Table 3 is a summary of the participant’s demographics and clinical characteristics.

**Table 3:** Participants characteristics

Participants Identification	Age	Occupation	Education	Marital status	Duration of infertility (years)	No. of living children	History of abortion	Time spent undergoing treatment (years)	Causes of infertility	Current treatment
P1	32	Employee	Bachelor	Married	4	No	No	2	F*	Surgery
P2	29	Entrepreneur	Diploma	Married	5	Yes (1)	No	3	M*	ART*
P3	28	Entrepreneur	Secondary school	Married	6	Yes (1)	Yes (2)	2	F	Surgery
P4	35	Employee	Bachelor	Living with a male partner	7	No	Yes (3)	4	F + M	ART
P5	32	Entrepreneur	Primary education	Married	6	Yes (1)	Yes (2)	3	F	Surgery
P6	35	Housewife	Secondary school	Married	8	No	No	4	F	Hormonal treatment
P7	34	Entrepreneur	Bachelor	Separated	6	Yes (1)	No	3	U*	Hormonal treatment
P8	42	Employee	Postgraduate	Married	12	Yes (1)	Yes (2)	8	F + M	ART
P9	28	Entrepreneur	Diploma	Married	3	No	No	2	U*	Hormonal treatment
P10	33	Housewife	Primary education	Married	6	No	Yes (1)	4	F	Hormonal treatment
P11	38	Employee	Postgraduate	Married	5	Yes (1)	No	3	F + M	ART
P12	21	Housewife	Secondary school	Married	2	No	No	1	F	Hormonal treatment
P13	44	Entrepreneur	Diploma	Divorce	14	Yes (1)	No	10	F	Surgery
P14	26	Housewife	Secondary school	Married	4	No	No	2	M	Varicocele surgical
P15	28	Entrepreneur	Diploma	Married	6	No	No	3	M	Varicocele Surgical
P16	42	Employee	Postgraduate	Separated	8	No	Yes (1)	6	F	Surgery
P17	33	Employee	Diploma	Living with a male partner	5	Yes (1)	No	2	F + M	ART
P18	29	Entrepreneur	Secondary school	Married	4	Yes (1)	No	2	F	Hormonal treatment
P19	32	Employee	Bachelor	Married	5	No	No	3	F	Surgery
P20	34	Entrepreneur	Primary education	Separated	6	No	No	3	M	Varicocele surgical
P21	35	Entrepreneur	Secondary school	Living with a male partner	7	Yes (2)	No	3	F	Hormonal treatment
P22	38	Housewife	Secondary school	Married	10	No	No	7	F + M	ART
P23	24	Entrepreneur	Secondary school	Married	3	No	No	1	F	Hormonal treatment
P24	33	Employee	Diploma	Married	5	Yes (1)	No	2	M	Varicocele surgical
P25	31	Housewife	Diploma	married	4	No	No	3	F	ART
P26	29	Employee	Diploma	Living with a male partner	3	No	Yes (1)	1	F	Surgery
P27	36	Employee	Diploma	Married	4	Yes (1)	No	3	F	ART
P28	34	Employee	Diploma	Separated	8	No	No	6	F	Surgery
P29	32	Housewife	Secondary school	Married	6	No	No	2	M	Varicocele surgical
P30	35	Employee	Secondary school	Divorced	7	No	No	5	U	Surgery
P31	28	Entrepreneur	Diploma	Married	4	Yes (1)	Yes (3)	3	U	ART
P32	36	Employee	Secondary school	Married	5	Yes (1)	No	3	F	Hormonal treatment
P33	40	Employee	Postgraduate	Married	6	Yes (1)	Yes (2)	4	U	ART

*F Female; M* male; U* unexplained; ART* Assisted reproductive technology (ART) included in-vitro fertilization (IVF) or intracytoplasmic sperm injection (ICSI)

## Themes

From the data analysis of the transcripts, three themes and 13 sub-themes were generated and are presented in Table 2. We present each theme below with subthemes, codes, and associated quotes.

### Theme 1. Challenges encountered during fertility treatment

This was the first theme to be identified from the data. Within this theme, three sub-themes were identified. These subthemes describe specific ways fertility treatment posed a challenge to women. According to women, fertility treatment is emotionally distressing, physically painful, and financial constraining.

#### Emotional distressing

All women experienced emotional distress and suffered from anxiety, worry, depression, and loneliness. One woman described the fertility treatment experience as passing through the desert, where there is nothing to drink or eat, only scorching sun, meaning that the treatment journey is torturous and accompanied by psychological suffering.

“I feel like I am passing through dessert, in a dessert, there is no water and something to eat, only scorching sunny, which burns, that how I feel, my heart is breaking and I am in escorting pain” (participant 30–35 years old-no child).

Most women reported that they were anxious going through the treatment process and particularly about whether or not they will achieve pregnancy.

“This is my eighth year in treatment; I have tried all other methods such as surgery and hormonal therapy and failed. I am now trying ART; we are worried that if I do not conceive now, it may be our last chance to be biological parents of another child” (participant 8–42 years old-1 child).

“My husband and I were very hopeful at the beginning of the treatment, but the doctor explained that we have only a 50% chance of getting a baby; it is like a gabble. I am always worried and anxious about the treatment outcome” (participant 11–38 years old-1 child).

Most women talked about being worried and concerned about their current age because they were scared of reaching pre-menopausal without having their child and therefore needed help.

“I am worried that I am aging and will be a pre-menopausal woman soon; I heard that a woman's fertility declines with age, so I came to the hospital to get help before it is too late” (participant 32–36 years old-1 child).

*It has been ten years since we started trying to get pregnant, we are worried that we are getting old without a baby, and I came to this hospital to see if they can help me* (participant 22–38 years old-no child).

Most women talked about the intense feeling of sorrow, disappointment, loss, and hopelessness following unsuccessful treatment.

“It is depressing; I have visited different hospitals and gynecologists and undergone surgery to remove a cyst on the left of my ovary. Now I am going through an IVF. I have spent much money seeking the solution, all in vain. Very disappointing” (participant 25–31 years old-no child).

“I was so disappointed last month after unsuccessful treatment. My husband and I spent a lot of energy and effort with no fruit. We went through a lot of pain and hurt; it was challenging for us because we started with a lot of hope but ended up with nothing.” (participant 4–35 years old-no child).

Most women reported profound sadness, frustration, despair, and anger during their menstruation, with some perceiving their periods as a loss of embryos, especially those undergoing ART. In addition, all women who had experienced miscarriages during infertility treatment reported feeling like they had lost a child and its relationship, leaving them in intense emotional pain.

“The doctor told me to wait for three weeks for embryo implantation to occur, then two weeks later, I got bleeding that how I lost my baby, to be honest, I was in shock and could not believe it, to say the least” (participant 32–36 years-1 child).

“Having a fertility issue is a problem on its own, and losing the baby, you have worked for hard for, you feeling like you are bleeding inside, I lost my IVF baby (embryo) at eight weeks” (participant 33–40 years old-1 child).

Most women interviewed described their infertility treatment experience as lonely and isolating because they lacked communication with their husbands and lost most of their friends.

“My husband does not want to talk about infertility or treatment; he keeps quiet or changes the topic when I try. I also lost most of my friends, and I had nobody to talk to except God” (participant 1–32 years old-no child).

“Going through fertility treatment is a painful and lonely experience it takes a lot from you, starting from husband, time, money, and friends, and many people do not have any idea how it feels like” (participant 7–34 years old-1 child).

Most women reported experiencing emptiness, defectiveness, incompleteness, unworthiness, and less of a woman.

“Treatment process hit hard; it leaves one feeling like the body is has a defect, incomplete and less of a human being leave alone a woman” (participant 19–32 years old-no child).

“Why me? What is wrong with my body? Why can I not be like other women who get pregnant easily? What did I do and did not do right, so I have to suffer like this by taking these drugs to get a baby? I feel like my body has failed me, and I empty from within” (participant 23–24 years old-no-child).

“Sometimes, I doubt my femininity. I say I am a woman who is a woman if she cannot conceive and give birth like other women. I have to get pregnant with the help of fertility treatment, and even with this assistance, I still cannot” (participant 6–35 years old-no child).

Notably, two women interviewed talked about having suicidal thoughts while pursuing treatment, although none had attempted suicide.

“I was driving from the hospital after my appointment; then a thought came to my mind, you useless woman, you may try to get the best care for your inability to have a baby, but no matter what, you will never have a baby. You are better off dead than alive because you have no value to the society, family and husband” (participant 28–34 years old-no child).

To conclude, women experienced various forms of psychological distress while seeking fertility treatment ranging from anxiety, worry, depression, loneliness, disappointment, and suicidal thoughts.

#### Physical pain

Almost all women reported experiencing physical pain during their fertility treatment, resulting from daily injections, intrusive procedures, and side effects of medications. The recommended drugs for infertility treatment include the gonadotrophin-releasing hormone (GnRH) to prevent premature ovulation, follicle-stimulating hormone (FSH) to stimulate ovarian follicle growth, human chorionic gonadotrophin (HCG) to facilitate the final maturation of oocytes, and progesterone to support the changes in the endometrium. These drugs are either administered orally or through injections. Frequent blood tests and transvaginal ultrasounds are needed to monitor the ovarian response. Some women described the treatment process as the following:

“The ART process is tormenting and traumatizing in general. For example, progesterone shots are painful as they must be injected deep into the muscle because they are oil-based. In addition to that, I have to get frequent blood tests, my whole body is full of pinholes, but I have to bear it since I want to have my baby” (participant 22-38 years old-0 child).

“The treatment process is torturous and punishing, especially going to the hospital daily for those injections. I had to juggle between hospital and work. I would go home very tired.” “(participant 17-33 years old-1 child).

“Frequent scans are a little annoying; I wish if they a way they can reduce them; it could be better. Egg retrieval was the toughest and most painful procedure. After the procedure, I had severe vomiting and could not eat anything. Both sides of my ovaries were swollen and sore, making it difficult to sleep and walk” (participant 25–31 years old-no child).

Almost all women reported having physical discomfort from hormonal therapy, such as weight gain, breast tenderness, ovarian pain, abdominal discomfort, and bloating. Some women expressed concern about the long-term effects of using fertility drugs.

“I experience severe bloating and headache after taking those fertility drugs, which is very uncomfortable” (participant 9–28 years old-no child).

“Since I started using the fertility drugs one year ago, I have gained 5 kg and frequently experience severe breast and stomach pain” (participant 12–21 years old-no child).

“I am worried about continuing using these drugs because we can never be sure if they can cause other diseases like cancer. I might be harming my body; you never know” (participant 21–35 years old-2 children).

In summary, women reported physical pain due to multiple injections, invasive procedures, and hormonal drugs related side effects while undergoing fertility treatment.

#### Financial constraining

All women, irrespective of their education and occupation, described fertility treatment as an expensive adventure. According to the women, many could afford the treatment because they had taken bank loans or borrowed from friends, sold their properties, supported by their husbands, and paid for by National Health Insurance Fund (NHIF) Card.

“I had to apply for a loan to pay for my treatment; I cannot afford it. For example, when I visit the hospital for an appointment, I need to pay more than KSH 5,000 for doctor’s consultation and prescribed drugs” (participant 10–33 years old-1 child).

“Fertility treatment is expensive, especially when surgery is involved. Last month, we had to ask our family and friends for money to pay for my fibroid removal operation” (participant 5-32 years old-1 child).

“We have spent all our savings on treatments, and we are now thinking of selling our car to cater for treatment” (participant 14–26 years old-no child).

One woman whose husband was paying for her treatment stated that her husband had been urging her to stop treatment so that they could save that money for other things.

“I know it is not easy for my husband; he works two jobs so I can undergo treatment, and we have our biological child. He wants me to give up the treatment so that we save money to buy a house, but that is not me. I consider a house useless; I cannot compare it with a baby” (participant 4–35 years old-no child).

Notably, all women who had experienced failed treatment, especially those paying the medical bill out of their pockets, reported feeling financial loss from treatment costs, transport costs, accommodation costs, time spent during treatment, and loss of jobs.

“What a loss. We sold our house to cater to the treatment cost; then, the treatment did not work. All the time and money we used for transport and accommodation to come to Nairobi is gone. It was a total loss” (participant 17–33 years old-1 child).

“I quit my job to concentrate on the infertility treatment because it was so exhausting, then after treatment what did I get a negative test, so heartbreaking” (participant 25–31 years old-no child).

Three women employees of the Kenya government talked about using their NHIF to pay for their fertility treatment. They still weighed that they could not have afforded the cost of treatment if not for the NHIF card.

“I can access the treatment without financial difficulty because I have an NHIF card; without it, I cannot afford it on my own” (participant 16–42 years old-no child).

“Fertility treatments are expensive, and most infertility treatments are not covered by medical insurance. It would not have been easy financially to access the treatment without this NHIF card” (participant 27–36 years old-1 child).

In summary, women reported that fertility treatments were expensive, and most experienced financial burdens during the treatment period. The study observed that some women funded their treatment, others were supported by their husbands, and others took loans or borrowed from friends and relatives. It was, therefore, essential to explore how fertility treatment affected women’s relations with their husbands, family members, and friends.

### Theme 2. Impacts of fertility treatment on relationships

The impact of fertility treatment on relationships was the second theme identified from the data. Within this theme, three subthemes were identified. These subthemes describe specific ways fertility treatment impacted their relationships. According to women, fertility treatment has negatively affected their relationships with their husbands, family members, and friends.

#### Relationship with their husband

Most women indicated that undergoing fertility treatment has negatively impacted their relationship with their husbands. According to women, one of the most affected aspects of their life was their sexual life. Some women reported that their husbands had lost sexual interest in them.

“Since he (my husband) started the treatment,  he lost interest in me sexually; he does not want anything to do with me” (participant 20–34 years old-no child).

“Honestly, I do not know what happened to him (my husband) when I started the treatment. He is no longer excited as he used to be sexually, always giving excuses; I feel tired. It is very frustrating. I feel like I am forcing him to do it” (participant 26–29 years old-no child).

Some women also indicated the process of infertility treatment had shifted sex from romantic and intimate to stressful events.

“Our intimate moment died after my husband started treatment; we only concentrate on the mechanical–biological aspect of it conceiving. I miss those days when we would have spontaneous sex without worrying about getting pregnant; that was pleasurable” (participant 24–33 years old-1 child).

“Since I started treatment, we have been scheduling sex during ovulation; my partner complains that he feels like a sperm donor” (participant 18–29 years old-1 child).

Some women reported that their husband’s attitude and behavior had changed after starting the fertility treatment ranging from verbally abusive, absenteeism, drug abuse, and not providing financially for their family.

“My partner no longer pays for anything in this house; when I ask, he says I do not have money; I use all of it on those stupid hospital bills. If you would have a child normally, I will not need to pay for it, so keep quiet. It hurts so deep” (participant 28–34 years old-no child).

“He (My Husband) started drinking excessively and became verbally abusive. He says I am useless, my work is to eat and misuse his money from one doctor to another, and I should go back to my father’s house if I cannot give him another baby” (participant 3–28 years-1 child).

“My husband became a work alcoholic; he started going to work very early in the morning, coming home late, and even working during the weekends. He does not want anything to do with the treatment process” (participant 13–44 years-1 child).

None of the women, however, mentioned physical abuse.

Only four women reported that the treatment process had positively contributed to their relationship with their husbands.

“During this treatment period, we have become more emotionally connected. We share our fertility treatment struggles. My husband understands me, and I understand him. He is more than my husband, my best friend, if I may say” (participant 31–28 years-1 child).

“I knew my husband cared for me but not to the extent he has shown me during this treatment period. He says kind words and is always there for me even if I feel like giving up. The treatment struggles have brought us together emotionally. We can comfortably share our difficult emotions without fear of being judged but with the attitude of being accommodated with understanding and love” (participant 33–40 years-1 child).

According to women, fertility treatment had given some couples hopes of having their biological child, and this hope rejuvenated their marriage.

“After many years of trying various kinds of treatment such as fertility treatment and numerous surgeries, we were on the verge of giving up. Our doctor mentioned ARTs, indicating it is the best treatment for us; we feel hopeful again of being given another chance to become parents. We are delighted and grateful it is a matter of time now.” (participant 27–36 years-1 child).

“Pursuing treatment and following doctors’ orders make my husband and I feel hopeful that one day we will able to have a baby of our own” (participant 29–32 years old-no child).

#### Relationship with their family

Most women felt that infertility treatments negatively impacted their family members’ relationships. According to women, one of the severely affected relationships was the women’s relationship with their in-laws. Some women reported that due to treatment, financial and time constraints were unable to attend to family needs and functions.

“My mother-in-law is unhappy with me because we do not support her financially like before. We do not have money. All of it goes to fertility treatment. She thinks I am the one preventing her son from sending her money. Our relationship with her has become strenuous” (participant 2–29 years old-1 child).

“I can no longer attend family gatherings because most of the time I spend between hospital and workplace. I am always tired and feel sick due to fertility medications. Nobody in my Husband understands why I have suddenly changed apart from my husband. I hope this situation will not last for a long time. I wish our relationship with my in-laws would return as it used to be” (participant 8–42 years old-1 child).

Most women whose in-laws knew they were undergoing treatment reported feeling pressured to get pregnant. This pressure resulted from repeated intrusive questions such as what did the doctor say? Is everything okay now? And how is the treatment going? Which often inflicted emotional pain and forced these women to stay away from family gathering functions and not pick up in-law’s phone calls.

“I do not pick up my husband’s relatives’ phone calls; they want to know everything about the treatment and just because they once helped us pay the medical bill. They start with those questions. Is everything okay now? What did the doctor say was wrong? When do we expect the visitor (baby), or when are we coming to drink porridge? (customary, when a child is born, other women go to see the new baby, and the host offers them wheat porridge). Those questions hurt so much; they are too personal to be discussed with anyone” (participant 15–28 years old-no child).

“My Husband’s family always offers us advice, such as which hospital to go to, new drugs or positions to try, even which church to go to for payers. It is very irritating and upsetting because they think we have not done anything and others think we need to do more. I try to keep off from them” (participant 19–32 years old-no child).

Most women choose not to disclose their pursuit of fertility treatment to their families because of perceived pressure that would result from constant questions such as whether there is there still hope. Significantly, most women feared their children would be stigmatized if they happened to conceive as they would not be regarded as normal.

“We do not disclose any information about our treatment journey; otherwise, we will answer all families’ questions about how the treatment is going, which can be very annoying. We also do not want our children to be stigmatized in the family and society” (participant 17–33 years old- 1 child).

“We do not share any information regarding our infertility treatment journey; we want it to remain a private affair. Also, if we get children, hopefully, we want them to be regarded as normal children rather than test tube babies” (participant 4–35 years old- no child).

Only two women reported that their relationship with their in-laws remained the same after starting fertility treatment.

“My relationship with my in-laws is the same as before starting the treatment; they do not know we are pursuing fertility treatment, but they can see something is wrong. The in-laws frequently visit us in our home; we feel supported and loved” (participant 10–33 years old- no child).

“Our relationship with our in-laws has remained almost the same even after starting the treatment. My husband’s family has supported us financially, emotionally, and spiritually during our treatment journey. They are our source of strength. We feel truly blessed!” (participant 5–32 years old- 1 child).

#### Relationship with their friends

Most women indicated that infertility treatments had negatively impacted their relationships with their friends. According to women, their friends suddenly lost interest in them and started treating them differently immediately after learning about their fertility treatment. This reaction left the women feeling disappointed.

“I am very disappointed with my best friend. Since I told her that I was facing some problems while seeking infertility treatment, she started treating me differently and lost interest in me. She stopped calling or texting me, visiting my home, and stopped inviting me to her children’s birthdays” (participant 1–32 years-no child).

“My friends lost interest in me and did not want to be associated with me after news spread that I had been spotted attending a fertility clinic in a nearby hospital. I feel hurt, sad, and deeply disappointed” (participant 9–28 years-no child).

Some women reported that they had lost a lot of friends during their infertility treatment journey. Some reasons for this loss were not getting financial support and lack of engagement with friends due to treatment constraints such as time and money.

“When I reached out to my friends for financial assistance to pay for my fertility test and treatment, they all disappeared, is when one is in problems knows their true friends. I am afraid to say I have no true friend now” (participant 2–29 years-1child).

“I have lost a lot of friends during this treatment period. One reason is I do not engage with them as I used to. I am always tired and feeling sick due to fertility treatment. I take breaks and rest more often than before. The second reason is that I do not have the money to visit new places or buy new stuff. I use that money to pay for the hospital bill and buy drugs. To treat infertility is an expensive affair in this country” (participant 12–21 years-no child).

One woman refused to talk about how fertility treatment has affected her relationship with friends. While looking down, she said:

“There are no friends in this world. I do not like talking about friends. I find no value in discussing having friends and speaking of people who will discourage you from seeking fertility treatment. God is the only true friend” (participant 26–29 years-no child).

Some women reported how asking intrusive questions about fertility treatment had impacted their relationship with their friends, particularly those who had helped raise money to pay the medical bills, which most participants did not appreciate.

“Friends ask many questions such as how is the treatment? And what do the doctors say about your progress? They repeatedly ask because they feel they have the right to ask questions and get answers. After all, they contributed to the hospital bills. It is tiring and frustrating to answer these types of questions” (participant 30–35 years-no child).

“I dislike speaking or answering questions about my infertility treatment struggle. So I keep off from friends who like asking such questions” (participant 20–34 years-no child).

Throughout fertility treatment, women face numerous challenges, including emotional distress, physical pain, and financial constraining. Women considered fertility treatment torturous and punishing, and oocyte retrieval was the most challenging procedure than injections, tests, and embryo transfer. The menstruation period was characterized by profound sadness, despair, and anger, especially for those women undergoing ARTs. Those women who had experienced a miscarriage during the treatment reported intense emotional pain from the perceived loss of a child and its relationship. Moreover, fertility treatment made women endure the negative impact of the relationship with their husbands, family, and friends. Therefore, it was essential to explore how these women coped with all these fertility treatment challenges.

### Theme 3. Coping with fertility treatment

From the data, there were six ways women tried to cope with the fertility treatment burden ranging from religious practices and personal faith, giving in to feelings, shifting focus, taking a break, staying with their relatives’ children, and receiving support from others.

#### Religious practices and personal faith

All women reported relying on their faith or religious practices to cope with fertility treatment challenges. The women described how reading the Bible, praying, and believing in God have been their source of strength as they pursued fertility treatment.

“If not for prayers and reading the word of God, I do not know how I could have managed to overcome the treatment burden. It is not easy, but my husband and I are coping well with the process through God’s grace” (participant 3–28 years old-1 child).

“I do not get very stressed by fertility treatment outcome because I believe Allah will give a child at His own time” (participant 13–44 years old-1 child).

“Knowing that in the Bible, Sarah, Hannah, and Elizabeth struggled with infertility, God answered their prayers. I get encouraged to continue with treatment. Knowing that the same God responded to their payers is the same God who will answer my prayers one day. When I remember that God is not a man and that He should lie, I stop worrying and wait on Him patiently” (participant 6–35 years old- no child).

#### Giving into feelings

Most women reported coping with fertility treatment challenges by crying, especially during menses.

“During my periods, I cannot hold my feelings, I cry and cry, but it makes me feel better equipped to deal with negative treatment outcomes. Although it is very frustrating” (participant 8–42 years old- 1 child).

“I cry almost every night because of fertility treatment emotional challenges. I think I cannot hold my feelings for long. After crying, I feel relieved and much better” (participant 21–35 years old- 2 children).

Some women said that they neglect doing their daily activities such as house cleaning, work, or even sleeping the whole day.

“When I get stressed about the treatment process, I do not do anything, even taking a shower or eating. I sleep for several days, and somehow, I regain my strength. Fertility treatment is a tough journey to be in” (participant 7–34 years old–1 child).

“I lost my business because I could not open it consistently because of the physical and emotional turmoil I was experiencing during the treatment process” (participant 18–29 years old-1 child). (participant 22–38 years old- no child).

Other women reported indulging themselves by buying new outfits and bags or going for dinner.

“When I feel depressed or sad during the treatment, I take myself out for dinner. It makes me feel special and like I am giving myself the attention I need” (participant 16–42 years old- no child).

“I buy new outfits and bags when I feel overwhelmed by the fertility treatment process. At least it makes me take my attention away from the treatment, even if it is for a while.” (participant 23–24 years old- no child).

#### Shifting focus

Some women reported coping with fertility treatment challenges by emotionally distancing themselves from the infertility treatment struggles. This distancing was done by keeping themselves busy with their career-related activities or getting involved in community services, which kept them busy, so they momentarily forgot about their fertility treatment problems.

“When I was started with infertility treatment, I decided to pursue higher education; I have done bachelor’s degree, last year I graduated with master’s degree. School work has kept me busy, making me forget temporarily about my treatment problems” (participant 16–42 years old-no child).

“Since I started the treatment process, I became more involved in voluntary work at church; it makes me useful and feels like I am giving back to society. Also, it takes my mind off fertility treatment-related challenges” (participant 28–34 years old-no child).

#### Taking a break

Some women reported that they coped with fertility treatment challenges by temporarily taking a break from treatments because treatments were emotionally, physically, and financially overwhelming.

“Last year, my husband and I decided to finish doing the infertility tests that the doctor ordered, then take a break for some months to rest as we look for money to do laparoscopy” (participant 32–36 years old-1 child).

“My husband and I just resumed fertility treatment last month. We had taken a break for two years because the treatment was emotionally challenging. We now feel ready to continue with treatment” (participant 29–32 years old-no child).

#### Staying with their relative’s children

Most of the women reported staying with their relative’s children. According to women, reasons for staying with the relative children, especially during the treatment process, included children giving them company, helping them with home chores, and creating a feeling of a complete family.

“I stay with two of my nieces, age 8 and 10 years. Those girls give me company as fertility treatment is a very isolating process. When I am not feeling so well, and my husband is not in the house, I can ask them to do simple chores like going to the shop to buy something.” “(participant 1–32 years old-no child).

“I would love to have twins, baby boys. Having my nephews in my house during treatment creates a complete family feeling because I care for them as their biological mother. I am a mother of two twin boys in waiting.” “(participant 15–28 years old-no child).

Interestingly, none of the women had adopted a child; when asked why most of them said they were not ready yet.

“Adopting a child is a critical decision a couple has to make. My husband and I are not yet ready for that now. If that time comes and we feel ready. We will be open to that option also. For now, we are pursuing the treatment” (participant 14–26 years old-no child).

“For a couple to adopt a child, they need to be prepared, more importantly psychologically, so that the child can grow in a loving and caring environment. We feel we are not ready yet but are open to it in the future. We are concentrating our efforts and time on seeking medical assistance” (participant 29–32 years old-no child).

#### Receiving support from others

Only a few women reported receiving financial, emotional, and spiritual support from their husbands, families, and friends while seeking fertility treatment.

“During the hospital appointment, my husband always accompanies me; he is my great source of support” (participant 33–40 years old-1 child).

“My mother is very supportive; she advises me a lot, encourages me to try new types of treatments, and prays for me” (participant 27–36 years old-1child).

“My friend, may God bless her, she has been supporting me all through my treatment journey, she has been helping me with my work assignment; she is such a good friend, caring, understanding, supportive and encouraging” (participant 11–38 years-1 child).

“My friends are always there for me. When I cry, they cry with me. They understand me even if they do not have fertility problems. They accompany me in my appointments and are the best friends one can ever need. I feel truly blessed to be my friend” (participant 5–32 years old-1 child).

## Discussion

This study explored the experiences of infertile women pursuing treatment and how they coped with fertility treatment challenges. The study found that women described fertility treatment as emotionally distressing, physically painful, and financially constraining. The participating women described the impact of fertility treatment on their relationships, including their relationships with their husbands, family members, and friends. They also identified six strategies they have used to deal with fertility treatment: religious practices and personal faith, giving in to feelings, shifting focus, taking a break, staying with their relatives’ children, and receiving support from others.

The reported experiences of emotionally challenging infertility treatment are consistent with previous study findings [21, 22]. A qualitative study conducted in Ghana to explore the experiences of women seeking ARTs found that women were anxious, stressed, and frustrated [23]. Another study conducted in China to examine Chinese couples’ experience of IVF treatment found that all women experienced emotional pain and suffered from anxiety, frustrations, disappointment, and sleep disturbances [24]. Similarly, a study conducted in Iran to explore the experiences of women who had used ARTs for their current pregnancy found that women experienced fear and uncertainty during the treatment [25]. No study reported findings regarding the three infertility treatment modalities, hormonal therapy, surgery, and ARTs. Consequently, this finding fills this literature gap. The result provides valuable information to healthcare professionals and policymakers on needing psychological support programs as part of fertility treatment services.

Women in this study indicated that the ARTs procedure was tormenting, traumatizing, and punishing. Egg retrieval was regarded as the most challenging and painful procedure. Moreover, women on hormonal therapy experienced undesirable side effects such as weight gain, breast tenderness, and headaches. This finding is consistent with previous studies, which reported that the ARTs process was torturous, stressful, exhausting, and time-consuming [23, 24]. This insightful finding may help healthcare professionals to provide evidence-based care to women undergoing fertility treatment. To access fertility treatment, women in this study took bank loans, borrowed from friends, and sold their properties, making them financially distressed. This finding is similar to other African and non-Africa countries, which describe fertility treatment, particularly ARTs, as expensive and reserved only for the rich [23, 26]. Thus, providing NHIF cards for fertility treatment may ease the financial challenge.

As in other African countries, communities in Kenya regarded having children as a moral obligation to one’s family and society. Individuals without children and their families are considered a disgrace to the community [27]. Therefore, procreation is considered mandatory in these communities and not an option [28]. Not surprisingly, most women in the study experienced marital distress, strenuous relations with in-laws, and pressure to get pregnant, which resulted from constant intrusive questions, making them keep away from their husbands’ relatives. Moreover, most women choose not to disclose their pursuit of fertility treatment to their families because of perceived pressure and fear that their children would be stigmatized if they happened to conceive. Other studies supported this finding [5, 29]. However, some women reported that fertility treatment had positively contributed to their marital relationship and that fertility treatment has given them some hope and rejuvenated their marriage. This finding is similar to previous studies [23, 24].

Regarding how fertility treatment has impacted women’s relationships with their friends, most women expressed disappointment with their friends for lack of support or understanding in their fertility treatment struggles. Moreover, some women reported that some of their friends asked intrusive and insensitive questions regarding their fertility treatment which most women did not appreciate, which made them keep off their friends. This finding is consistent with the result of the Kamau study, which revealed that most married Kenyan women with infertility had a low opinion of their friends [30]. This finding indicates the need to educate the Kenyan society about infertility and its treatments. This education may lead to a better understanding of the experiences of infertile women seeking treatment in Kenya. Thus, reducing social stigma and women reconnecting with their families and friends who may provide them with social support.

Our study found that Kenyan women coped with the burden of fertility treatment by reading the Bible, praying, and believing in God. Some women reported they were able to cope through crying, neglecting their daily chores, indulging themselves, keeping themselves busy, taking a break from treatment, staying with relatives’ children, and receiving support from husbands, family, and friends. This finding is consistent with the results of other African studies [31, 32]. Interestingly, none of the women in the study had adopted a child. Similar to Mogobe’s [33] study, which found that infertile women thought adoption was an afterthought after a failed attempt to conceive biological children. This finding highlighted the need for mental health professionals to develop a comprehensive counseling program for infertile women that provides information on alternative ways of starting a family, such as adoption.

Our study had several limitations. First, we recruited participants from three referral hospitals in Kiambu and Nairobi counties. Second, only voluntary women who had access to a mobile phone were interviewed. There would have been more varied experiences if this study had included women seeking treatment in non-referral hospitals, non-voluntary and without mobile phones. Therefore, the study findings cannot generalize all Kenyan women pursuing treatment. Third, responses might have been influenced by social desirability bias. Future studies regarding infertility and its treatment are needed in Kenya. A specific research recommendation is to conduct a similar study with men pursuing treatment to explore their fertility treatment experiences. Another suggestion is to conduct a similar study with women seeking treatment in non-referral hospitals to examine their fertility treatment experience compared with themes identified in this study. Similarly, research involving health care professionals regarding fertility treatment may help Kenyan society begin to appreciate the experiences of infertile women pursuing treatment. Lastly, studies to examine ways to reduce the burden for women seeking treatment so that appropriate intervention can be developed for these women.

## Conclusion

This study was the first to explore the experiences of women seeking fertility treatment in Kenya. It was found that women described infertility treatment as emotionally distressing, physically painful, and financially constraining. They also reported how fertility treatment negatively affected their relationship with their husbands, family, and friends. In addition, women pointed out six coping strategies they used to navigate fertility treatment challenges, including religious beliefs, giving in to feelings, shifting focus, taking breaks, staying with their relative’s children, and receiving support from others. The findings offer insights into the plight of women seeking fertility treatment and the need for incorporating psychosocial interventions or counseling into the fertility treatment routine to help these women get through treatment challenges. The findings also provide an empirical contribution to the Kenyan government to provide NHIF cards to women undergoing fertility treatment. It is also suggested that healthcare professionals need to educate Kenyan society about infertility and its treatment to reduce social stigma. It is hoped that incorporating psychosocial interventions or counseling would reduce the fertility treatment burden for women undergoing treatment and be flexible on the issues of bearing children, improving their psychological well-being and relationships with their husbands, family, and friends.

## Data Availability

The datasets analyzed during this study are available from the corresponding author on reasonable request.

## Abbreviations

KNH: Kenyatta national hospital
NHIF: National health insurance fund
ARTs: Assisted reproductive technologies
IVF: In -vitro fertilization
ICSI: Intracytoplasmic sperm injection
GIFT: Gamete Intra Fallopian Transfer
TET: Tubal Embryo Transfer
ZIFT: Zygote Intra Fallopian Transfer
NACOSTI: National Commission for science, technology, and innovation
